# Concentration-Dependent Reinforcement of Self-Curing Poly(methyl methacrylate) with Polyetheretherketone: Mechanical Performance and Physicochemical Stability

**DOI:** 10.3390/ma19071320

**Published:** 2026-03-26

**Authors:** Hsiu-Na Lin, May-Show Chen, Wei-Fang Lee, Pei-Wen Peng, Tzu-Yu Peng, Tien-Li Ma, Chung-Kwei Lin

**Affiliations:** 1 Research Center of Digital Oral Science and Technology, College of Oral Medicine, Taipei Medical University, Taipei 11031, Taiwan; tiffanylin1214@gmail.com (H.-N.L.); maychen@tmu.edu.tw (M.-S.C.); typeng@tmu.edu.tw (T.-Y.P.); 2 Department of Dentistry, Chang Gung Memorial Hospital, Taipei 10507, Taiwan; 3 School of Dentistry, College of Oral Medicine, Taipei Medical University, Taipei 11031, Taiwan; 4 Division of Prosthodontics, Department of Dentistry, Taipei Medical University Hospital, Taipei 11031, Taiwan; 5 School of Dental Technology, College of Oral Medicine, Taipei Medical University, Taipei 11031, Taiwan; weiwei@tmu.edu.tw (W.-F.L.); apon@tmu.edu.tw (P.-W.P.)

**Keywords:** poly(methyl methacrylate), polyetheretherketone, denture base resin, mechanical properties, water sorption, physicochemical stability, dental biomaterials

## Abstract

**Highlights:**

- PEEK particles improved flexural and compressive strength of PMMA.
- Mechanical reinforcement showed concentration-dependent behavior.
- Water sorption and solubility remained within ISO limits.
- Reinforced PMMA may enhance interim prosthesis durability.
- Optimal filler concentration balances strength and stability.
- Provides a practical modification strategy for chairside materials.

**Abstract:**

Self-curing poly(methyl methacrylate) (PMMA) remains widely used for provisional restorations and denture bases; however, its limited mechanical strength and susceptibility to water-related degradation restrict long-term performance. This study investigated the concentration-dependent reinforcement of self-curing PMMA with polyetheretherketone (PEEK) particles and evaluated mechanical properties and physicochemical stability. PMMA specimens containing different PEEK concentrations were fabricated and tested for flexural strength, compressive strength, surface hardness, water sorption, and water solubility according to standardized protocols. Mechanical performance demonstrated a concentration-dependent enhancement, with moderate PEEK incorporation significantly improving strength parameters compared to the control group. Excessive filler loading, however, did not yield proportional improvements. Water sorption and solubility values remained within clinically acceptable and ISO-recommended limits. These findings suggest that controlled PEEK reinforcement provides a feasible approach to enhancing the mechanical durability of self-curing PMMA without compromising physicochemical stability. The study offers a practical material modification strategy for improving interim prosthetic materials in clinical dentistry.

## 1. Introduction

Poly(methyl methacrylate) (PMMA) remains one of the most widely used polymeric materials in dentistry, particularly for denture bases, provisional restorations, and relining applications due to its ease of manipulation, acceptable aesthetics, and cost-effectiveness [1,2,3]. Self-curing PMMA systems are especially valuable in chairside procedures because of their rapid polymerization and clinical convenience. PMMA-based materials constitute a significant proportion of dental prosthetic and restorative materials used worldwide, reflecting their extensive clinical demand and economic importance [4]. However, despite these advantages, conventional self-curing PMMA exhibits intrinsic limitations including relatively low flexural strength, poor impact resistance, and susceptibility to water-induced plasticization and hydrolytic degradation, which may compromise mechanical durability and dimensional stability during service [5,6,7]. These shortcomings are particularly critical in interim prosthetic applications, where materials are subjected to repetitive functional loading and continuous exposure to saliva and thermal fluctuations.

To improve the mechanical reliability of PMMA, various reinforcement strategies have been explored, including the incorporation of fibers, inorganic nanoparticles, and ceramic fillers [8,9,10,11]. Reinforcement mechanisms in polymer composites typically involve load transfer from the ductile matrix to stiffer filler phases, restriction of polymer chain mobility, and crack deflection or bridging effects. Nanofillers such as zirconia, TiO_2_, graphene oxide, and nanodiamonds have demonstrated improvements in flexural strength and surface hardness [11,12,13]. Nevertheless, several challenges remain. Inorganic fillers may suffer from poor interfacial adhesion with the PMMA matrix, leading to stress concentration sites and interfacial debonding under load. Furthermore, particle agglomeration at higher filler concentrations can reduce reinforcement efficiency and negatively affect mechanical performance [2,3]. Excessive filler loading may also disrupt matrix continuity, impair polymerization kinetics, and increase water sorption or solubility due to interfacial void formation.

Polyetheretherketone (PEEK) is a high-performance semicrystalline thermoplastic polymer characterized by excellent mechanical strength, chemical resistance, thermal stability, and biocompatibility [14,15,16,17]. Owing to its favorable elastic modulus and resistance to hydrolytic degradation, PEEK has gained increasing attention in dental applications, including removable prostheses, implant components, and fixed restorations [14,16,18]. Compared with ceramic or metallic fillers, PEEK offers potential advantages as a reinforcing phase in PMMA systems because of polymer–polymer compatibility. The chemical affinity between PEEK and PMMA may promote improved interfacial stress transfer and reduce modulus mismatch relative to inorganic fillers. In addition, the relatively low water uptake of PEEK may contribute to enhanced physicochemical stability when incorporated at appropriate concentrations.

Despite these promising attributes, most previous investigations have focused on bulk PEEK applications, fiber-reinforced PEEK systems, or high-loading composite structures [17,19,20]. Systematic investigations on concentration-dependent particulate PEEK incorporation into self-curing PMMA remain scarce. In polymer composite systems, reinforcement efficiency is highly dependent on filler content. At low concentrations, fillers may enhance stiffness and strength through effective stress transfer and restriction of polymer chain mobility. However, beyond a critical threshold, particle–particle interactions, agglomeration, and matrix discontinuities may counteract reinforcement benefits and introduce defects that adversely affect mechanical integrity and water-related stability. In recent years, nanomaterials have attracted increasing attention in dentistry due to their ability to enhance mechanical performance through nanoscale interactions and improved interfacial bonding [21,22]. These effects can significantly influence the overall behavior of polymer-based composites. However, nanofillers also present challenges, including particle agglomeration and difficulties in achieving uniform dispersion, which may adversely affect physicochemical stability. These limitations highlight the need for alternative reinforcement strategies that can achieve a balance between mechanical enhancement and material stability. In this context, polymer-based fillers such as PEEK offer a promising approach due to their compatibility with polymer matrices and more stable dispersion behavior. Therefore, identifying an optimal filler concentration is essential to achieving a balanced combination of mechanical reinforcement and physicochemical performance.

From a clinical perspective, provisional dental materials are continuously exposed to aqueous environments. Water sorption can induce plasticization and dimensional change, while solubility may lead to material degradation and surface deterioration. Moreover, color stability is a critical factor in aesthetic dentistry, particularly for interim restorations [23,24,25,26]. Therefore, evaluation of water sorption, solubility, and color stability is indispensable when assessing the feasibility of modified PMMA systems for clinical use.

Accordingly, the aim of this study was to investigate the concentration-dependent effects of particulate PEEK incorporation on the mechanical performance and physicochemical stability of self-curing PMMA. Mechanical properties including flexural strength, elastic modulus, and surface microhardness were evaluated, together with water sorption, solubility, and color stability under simulated aqueous conditions. The null hypothesis was that incorporation of PEEK particles would not significantly influence the mechanical or physicochemical properties of the PMMA matrix.

## 2. Materials and Methods

### 2.1. Materials and Specimen Preparation

A commercially available self-curing poly(methyl methacrylate) (PMMA) resin (UNIFAST Trad; GC Corp., Tokyo, Japan) was used as the matrix material to simulate chairside provisional and relining applications. Biomedical-grade polyetheretherketone (PEEK) powder (ICI Ltd., London, UK) was used as the reinforcing filler.

PEEK particles were incorporated into PMMA powder at 0 (control), 1, 3, 5, 10, 15, and 20 wt.%. The total powder mass per batch was fixed at 5 g to ensure consistency. The PMMA powder-to-liquid monomer ratio was maintained at 2:1 (g/mL) according to the manufacturer’s instructions to ensure consistent polymerization behavior and clinical relevance [23].

**Figure 1:**
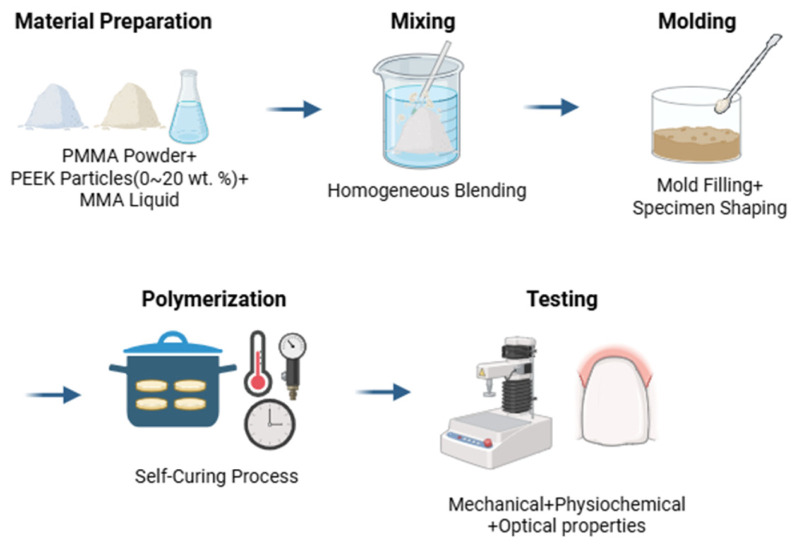
Schematic illustration of the experimental workflow, including PMMA–PEEK mixing, specimen fabrication, curing, and subsequent mechanical and physicochemical testing.

PEEK and PMMA powders were mechanically dry-mixed for 5 min using a laboratory mixer at 300 rpm to promote homogeneous dispersion prior to monomer addition. The monomer was then added and manually mixed for 30 s until a dough-like consistency was achieved. A schematic overview of the specimen preparation and testing workflow is presented in Figure 1 to facilitate understanding of the experimental procedures.

### 2.2. Specimen Fabrication

The dough-phase material was packed into custom silicone molds corresponding to each test geometry. A standardized compressive load of 1.5 kg was applied using a glass plate for 5 min to minimize porosity. Polymerization was carried out in a pressure-curing unit at 45 °C and 2 bar for 15 min. After curing, specimens were removed and inspected under ×10 magnification for voids or defects. Specimens with visible defects were discarded.

All specimens were sequentially polished using 600-, 800-, and 1200-grit silicon carbide papers under continuous water irrigation. Final dimensions were verified using a digital caliper (±0.01 mm accuracy). Dimensional tolerances were controlled within ±0.1 mm. Specimens were stored in distilled water at 37 °C for 72 h prior to testing.

### 2.3. Group Allocation and Sample Size

A total of 120 specimens were fabricated and distributed among the different experimental evaluations. For each PEEK concentration, five specimens (n = 5) were prepared for flexural testing, microhardness evaluation, water sorption and solubility assessment, and color stability analysis. The selected sample size was consistent with previous in vitro investigations of PMMA-based composite materials and was considered adequate for comparative statistical analysis.

### 2.4. Fourier Transform Infrared Spectroscopy (FTIR)

Fourier transform infrared spectroscopy was performed using an ATR-FTIR spectrometer (Frontier FTIR; PerkinElmer, Waltham, MA, USA) to confirm the chemical characteristics of the composite systems. Spectra were collected over a wavenumber range of 4000–600 cm^−1^ at a resolution of 4 cm^−1^ with 32 scans per specimen. Characteristic absorption peaks corresponding to PMMA (C=O stretching at approximately 1720 cm^−1^) and PEEK (aromatic ring vibration near 1590 cm^−1^) were analyzed to verify the physical incorporation of PEEK particles within the PMMA matrix and to detect potential chemical interactions.

### 2.5. Vickers Microhardness Testing

Surface microhardness was evaluated using a digital microhardness tester (TAI CHONG Co., Ltd., Taichung, Taiwan). Disc-shaped specimens measuring 10 ± 0.1 mm in diameter and 1 ± 0.05 mm in thickness were conditioned at 37 °C and 100% relative humidity for 72 h prior to testing. A load of 50 g was applied for 10 s using a diamond indenter. Three indentations were performed on non-overlapping regions of each specimen, with a minimum spacing of 1 mm between indentations to avoid interaction effects. The mean Vickers hardness (HV) value was calculated for each specimen.

### 2.6. Flexural Testing Procedure

Flexural properties were assessed using a universal testing machine (Sans Testing Machine Co., Ltd., Shenzhen, China) under a three-point bending configuration. Rectangular specimens measuring 25 ± 0.1 mm × 2 ± 0.1 mm × 2 ± 0.1 mm were tested with a support span of 20 mm and a crosshead speed of 0.5 mm/min. The maximum load at fracture (F) was recorded, and flexural strength (FS) was calculated using the equation:

FS = 3FL/(2WH^2^)

where L represents the span length (mm), W the specimen width (mm), and H the specimen thickness (mm). The elastic modulus was determined from the slope of the initial linear portion of the stress–strain curve.

### 2.7. Water Sorption and Solubility Measurement

Water sorption and solubility were evaluated in accordance with ISO 4049:2013 [27]. Disc specimens measuring 15 ± 0.1 mm in diameter and 1 ± 0.05 mm in thickness were first dried at 37 °C in a desiccator until a constant mass (*m*_1_) was achieved. Specimens were then immersed individually in 5 mL of deionized water and stored at 37 °C for 7 days. After immersion, specimens were gently blotted dry and weighed (*m*_2_), followed by re-drying to constant mass (*m*_3_). Water sorption (*W_sp_*) and solubility (*W_sl_*) were calculated using:

*W_sp_* = (*m*_2_ − *m*_3_)/*V*

*W_sl_* = (*m*_1_ − *m*_3_)/*V*

where *V* denotes specimen volume (mm^3^). ISO acceptance thresholds were defined as *W_sp_* < 40 µg/mm^3^ and *W_sl_* < 7.5 µg/mm^3^.

### 2.8. Color Stability Evaluation

Color measurements were performed using a spectrophotometer (OptiShade; StyleItaliano, Casarza Ligure, Italy) based on the CIELab color system. Baseline color values were recorded prior to storage. Specimens were subsequently stored in ambient air, deionized water, or simulated body fluid (SBF prepared according to the Kokubo protocol; confirm if used). Color measurements were obtained after 24 h, 7 days, and 28 days. Color differences (ΔE) were calculated using the standard CIELab equation. A ΔE value below 3.7 was considered clinically acceptable, in accordance with previously reported perceptibility thresholds [23,24].

### 2.9. Statistical Analysis

Normality of data distribution was assessed using the Shapiro–Wilk test. As several datasets did not meet the assumption of normality, nonparametric Kruskal–Wallis tests were performed to evaluate differences among groups, followed by Dunn’s post hoc multiple comparison tests where appropriate. Statistical analysis was conducted using SPSS software (version 25.0; IBM Corp., Armonk, NY, USA). Statistical significance was set at *p* < 0.05.

This study was conducted entirely in vitro and did not involve human participants or animal experimentation; therefore, ethical approval was not required.

## 3. Results

### 3.1. Fourier Transform Infrared Spectroscopy

**Figure 2:**
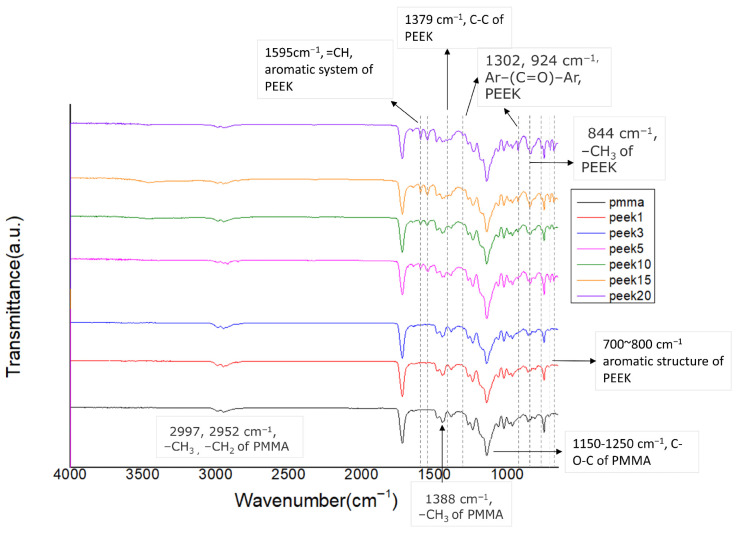
FTIR spectra of PMMA and PEEK-reinforced PMMA composites with varying PEEK contents (1–20 wt.%). Key absorption peaks corresponding to PMMA and PEEK functional groups are indicated.

The ATR-FTIR spectra of unreinforced PMMA and PEEK-reinforced composites are presented in Figure 2. The characteristic absorption peaks of PMMA, including the ester carbonyl stretching band at approximately 1720 cm^−1^ and aliphatic C–H stretching vibrations in the range of 2950–3000 cm^−1^, were observed in all groups. Upon incorporation of PEEK, additional absorption features associated with aromatic ring vibrations (1590 cm^−1^) and ether linkages were detected.

No new absorption bands or significant peak shifts were identified across the composite formulations compared with the parent materials. The spectra indicated the coexistence of PMMA and PEEK characteristic peaks without evidence of chemical modification.

### 3.2. Vickers Microhardness

**Figure 3:**
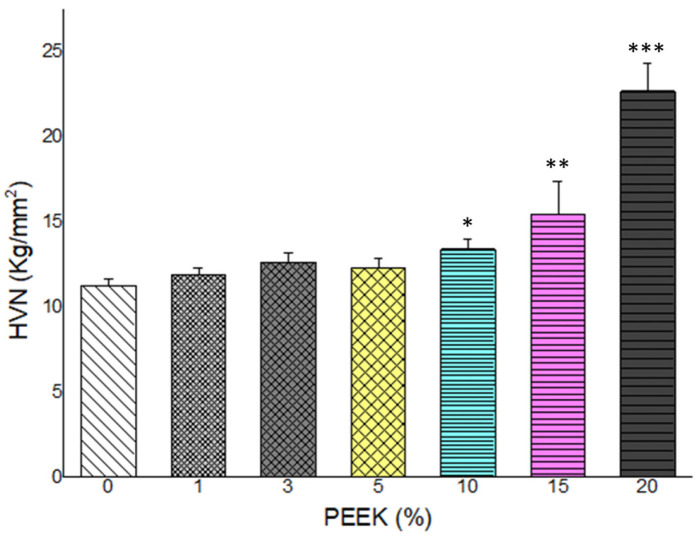
Vickers microhardness (HVN) values of PMMA composites reinforced with varying concentrations of PEEK (0–20 wt.%). A progressive increase in surface hardness is observed with increasing PEEK content, with the highest value recorded at 20% PEEK. (n = 5). Group comparisons were performed using the Kruskal–Wallis H test. (*: *p* < 0.05, **: *p* < 0.01, ***: *p* < 0.001).

Vickers microhardness values are shown in Figure 3. Surface hardness increased progressively with increasing PEEK content. All PEEK-containing groups exhibited higher hardness values compared with the control group. The highest microhardness was recorded in the 20 wt.% PEEK group.

Statistical analysis revealed significant differences among groups (*p* < 0.05). The increase in hardness demonstrated a concentration-dependent trend across the evaluated range.

### 3.3. Flexural Strength and Elastic Modulus

**Figure 4:**
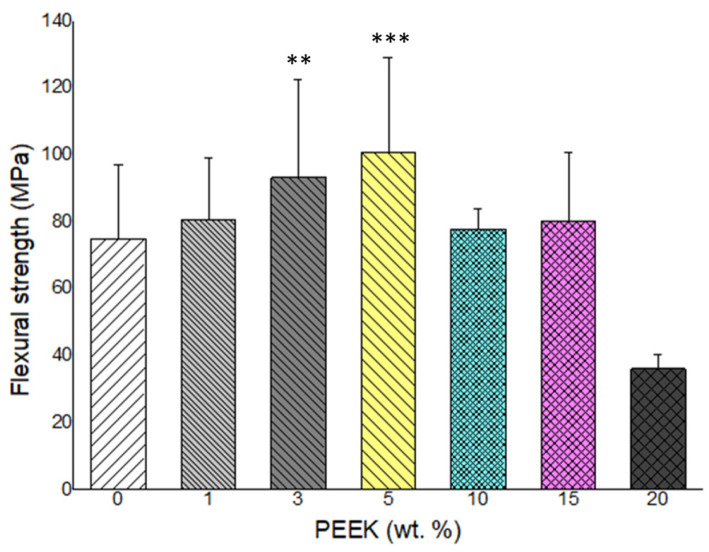
Flexural strength of PMMA composites containing 0% to 20% PEEK by weight. The highest strength was observed at 5 wt.% PEEK, with values decreasing at higher concentrations (n = 5) (**: *p* < 0.01, ***: *p* < 0.001).

**Figure 5:**
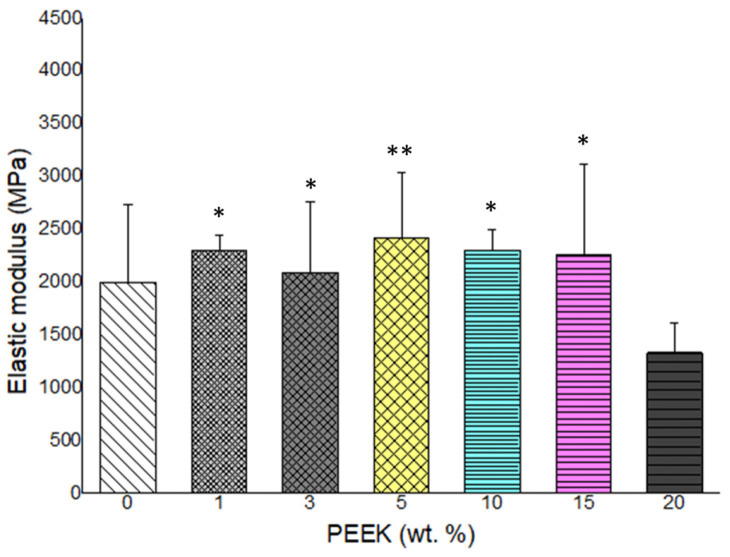
Elastic modulus of PMMA composites as a function of PEEK content (0–20 wt.%). A peak modulus was recorded at 5 wt.% PEEK, followed by a slight decrease at higher concentrations (n = 5) (*: *p* < 0.05, **: *p* < 0.01).

Flexural strength and elastic modulus results are presented in Figure 4 and Figure 5, respectively. Incorporation of low concentrations of PEEK resulted in increased flexural properties relative to the control group. The highest flexural strength and modulus were observed at 5 wt.% PEEK.

When the PEEK content exceeded 5 wt.%, both flexural strength and modulus decreased progressively. At 20 wt.% PEEK, flexural values were lower than those of the unreinforced control. Statistical analysis confirmed significant differences among groups (*p* < 0.05). The mechanical response exhibited a non-linear concentration-dependent behavior, with an initial enhancement followed by reduction at higher filler loadings.

### 3.4. Water Sorption and Solubility

**Figure 6:**
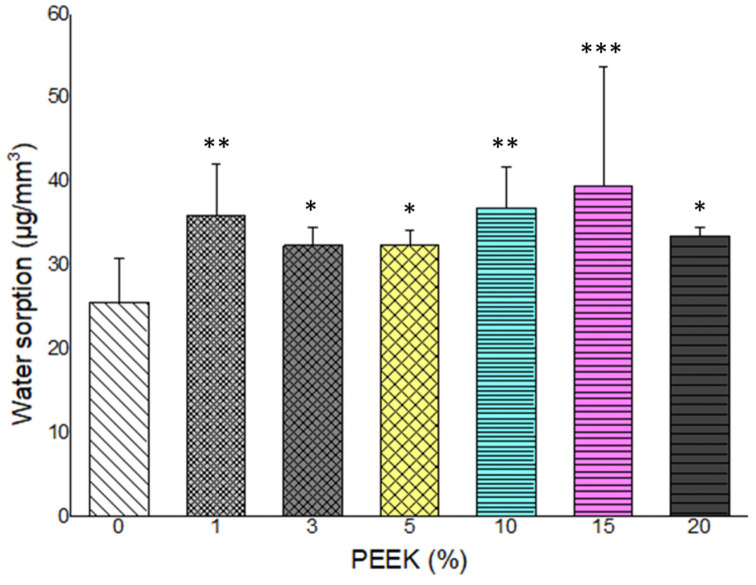
Water sorption of PMMA composites containing 0–20 wt.% PEEK. All groups remained within the ISO 4049:2013 standard limit of 40 μg/mm^3^ (n = 5). (*: *p* < 0.05, **: *p* < 0.01, ***: *p* < 0.001).

**Figure 7:**
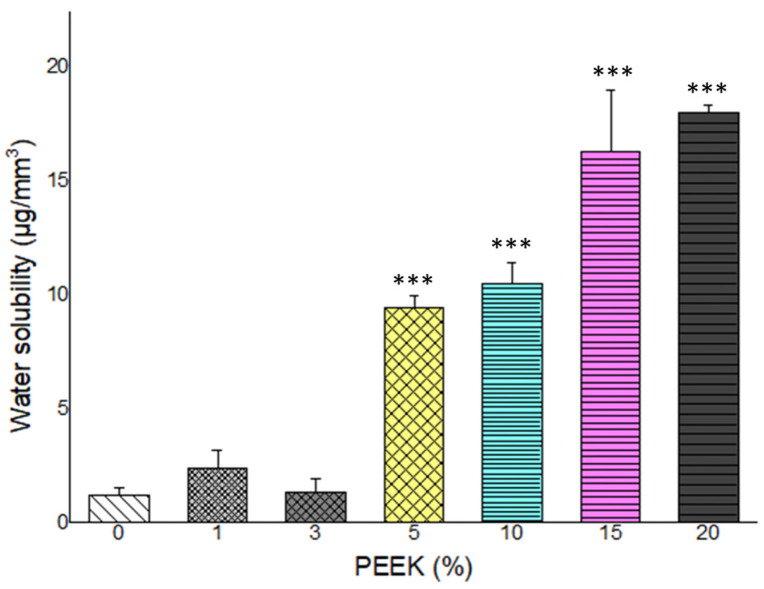
Water solubility of PMMA composites with varying PEEK contents. Solubility increased significantly beyond 5 wt.% PEEK, exceeding the ISO 4049:2013 threshold of 7.5 μg/mm^3^. (n = 5). (***: *p* < 0.001).

Water sorption and solubility results are presented in Figure 6 and Figure 7. All groups demonstrated water sorption values below the ISO 4049:2013 limit of 40 µg/mm^3^. A slight increase in water sorption was observed with increasing PEEK concentration; however, values remained within the standard threshold across all groups.

Water solubility showed a concentration-dependent increase. The control, 1 wt.%, and 3 wt.% PEEK groups met the ISO 4049:2013 solubility requirement (<7.5 µg/mm^3^). Groups containing 5 wt.% PEEK and above exceeded the recommended limit. The highest solubility values were recorded in the 20 wt.% PEEK group. Statistical differences among groups were significant (*p* < 0.05).

### 3.5. Color Stability

**Figure 8:**
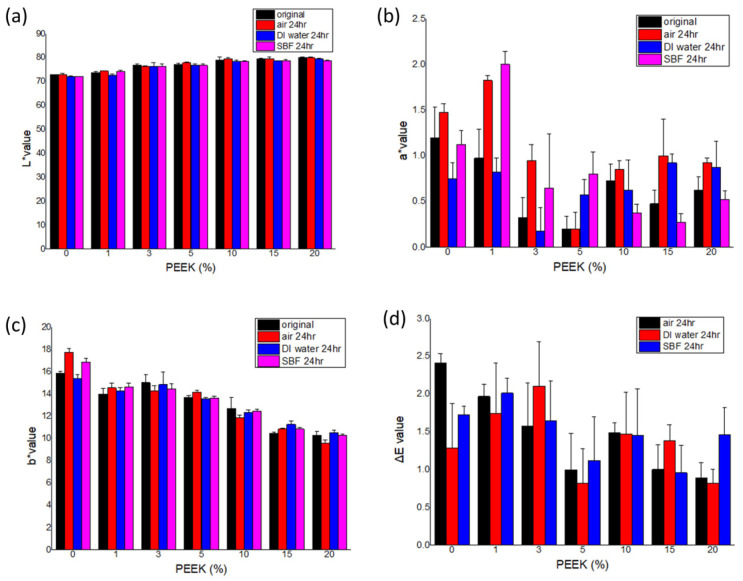
Color parameters of self-curing PMMA composites containing 0% to 20% PEEK after 24 h of storage in air, deionized water (DI water), and simulated body fluid (SBF). (**a**) L* values, (**b**) a* values, (**c**) b* values, (**d**) Color difference (ΔE) values. (n = 5).

**Figure 9:**
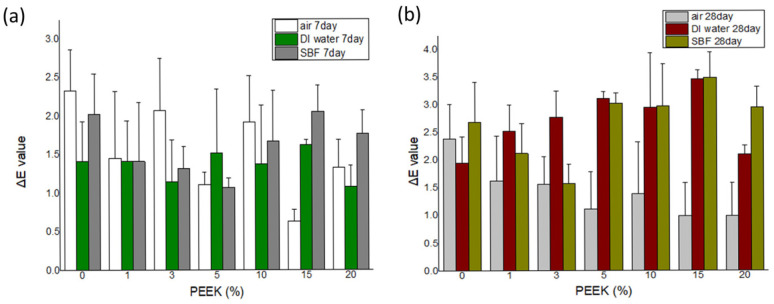
Color difference (ΔE) values of self-curing PMMA composites containing 0% to 20% PEEK after (**a**) 7 days, (**b**) 28 days of storage in three different environments: air, deionized water (DI water), and simulated body fluid (SBF). All ΔE values remained below the clinically perceptible threshold of 3.7, indicating acceptable color stability across all conditions (n = 5).

Color stability data are presented in Figure 8 and Figure 9. Across all storage conditions and evaluation periods (24 h, 7 days, and 28 days), ΔE values remained below 3.7 for all formulations.

Minor variations in L*, a*, and b* parameters were observed over time. Composites containing lower PEEK concentrations (0–3 wt.%) generally exhibited smaller ΔE values compared with higher concentrations; however, all groups remained within the perceptibility threshold.

ΔE values tended to stabilize after 7 days of storage across all media.

## 4. Discussion

The present study demonstrated a concentration-dependent reinforcement behavior of particulate PEEK incorporated into self-curing poly(methyl methacrylate). The mechanical response exhibited a non-linear trend, characterized by initial enhancement at low filler concentrations followed by deterioration at higher loadings [28,29]. Similar non-linear reinforcement behavior has been reported in PMMA-based composite systems containing inorganic fillers, where mechanical enhancement is observed only within a limited concentration range before agglomeration effects dominate [30,31,32]. The maximum PEEK content was limited to 20 wt.% due to considerations of processability, dispersion behavior, and matrix continuity. At higher filler loadings, preliminary observations indicated reduced workability and increased likelihood of particle agglomeration, which may adversely affect composite integrity and polymerization behavior [3,11].

At low PEEK concentrations (1–5 wt.%), improvements in flexural strength and elastic modulus were observed. Such enhancement can be explained by classical load transfer mechanisms in particulate-reinforced polymer composites. When filler dispersion is adequate, rigid particles restrict matrix deformation and facilitate stress transfer under applied load. Comparable strengthening trends have been reported in PMMA systems reinforced with zirconia nanoparticles and other nanofillers [10]. The absence of significant peak shifts in FTIR spectra suggests that reinforcement in the present system was governed by physical incorporation rather than chemical bonding, consistent with previous observations in filler-modified PMMA matrices [3].

The maximum flexural performance observed at 5 wt.% PEEK suggests the existence of an optimal filler threshold. Beyond this concentration, flexural strength and modulus decreased progressively. This phenomenon may be attributed to increased particle–particle interactions and reduced interparticle spacing, which promote agglomeration and stress concentration. Similar reductions in mechanical properties at elevated filler contents have been documented in PMMA composites lacking surface treatment or coupling agents [2,3]. In untreated systems, weak interfacial adhesion can lead to microvoid formation and inefficient stress transfer, ultimately reducing structural integrity. In addition, modification of the interfacial region using coupling agents, such as ethylene glycol dimethacrylate (EGDMA), may further improve interfacial adhesion and stress transfer efficiency between PEEK particles and the PMMA matrix. Such strategies could potentially mitigate the deterioration in mechanical properties observed at higher filler loadings and represent an important direction for future optimization.

The monotonic increase in microhardness with increasing PEEK content contrasts with the non-linear flexural behavior. This discrepancy reflects the difference between localized surface deformation and bulk load-bearing performance. Surface hardness is primarily influenced by the presence of rigid particles near the indentation site, whereas flexural strength depends on uniform filler dispersion and overall matrix continuity. Comparable divergence between hardness and flexural strength has been reported in nanofiller-modified PMMA systems [33].

Water sorption remained below ISO limits for all groups, indicating that incorporation of PEEK did not substantially alter overall moisture uptake. Given the relatively hydrophobic nature of PEEK, this observation is consistent with previous reports describing the favorable aqueous stability of PEEK-based materials [18]. However, water solubility increased significantly at filler contents ≥5 wt.%, exceeding ISO thresholds. Increased solubility at higher filler loadings has also been reported in PMMA composites containing inorganic fillers, where interfacial discontinuities facilitate leaching of residual monomers or low-molecular-weight components [26,34]. The divergence between acceptable water sorption and elevated solubility suggests that interfacial defects, rather than bulk water diffusion, may govern degradation at higher filler concentrations. It should be noted that different conditioning protocols were applied depending on the property evaluated. Mechanical properties and water sorption/solubility were assessed after standardized conditioning (37 °C for 72 h) to ensure consistency with established testing methods, whereas color stability was evaluated under multiple time points to reflect its sensitivity to environmental exposure. These differences in experimental design were intended to capture the most clinically relevant aspects of each property.

Color stability remained within the clinically perceptible threshold across all storage conditions. PMMA-based provisional materials are generally considered to exhibit acceptable short-term optical stability [35,36], and the chemical inertness of PEEK may further contribute to preservation of chromatic properties [18]. The stabilization of ΔE values after 7 days indicates that initial equilibration processes dominated the observed optical changes.

Taken together, these findings suggest that particulate PEEK functions as an effective reinforcing phase at low concentrations but exhibits diminishing returns beyond a critical threshold. The observed non-linear response reflects a balance between mechanical reinforcement and interfacial defect formation. Identification of an optimal concentration range is therefore essential to maximize performance while preserving physicochemical stability.

Several limitations should be acknowledged. While microstructural characterization techniques such as scanning electron microscopy (SEM) or transmission electron microscopy (TEM) could provide additional insight into particle dispersion and interfacial morphology, the present study was designed to focus on functional performance and clinically relevant physicochemical properties. The concentration-dependent trends observed in mechanical behavior and water interaction provide indirect but consistent evidence of filler distribution and matrix integrity. In addition, long-term hydrothermal aging and fatigue performance were not evaluated in this study. Future investigations incorporating advanced microstructural analysis, surface-treated PEEK particles, and extended aging protocols are warranted to further elucidate reinforcement mechanisms and improve the long-term durability of these composite systems.

## 5. Conclusions

This study demonstrated that incorporation of particulate polyetheretherketone (PEEK) into self-curing poly(methyl methacrylate) (PMMA) resulted in a concentration-dependent reinforcement effect with a non-linear mechanical response. Flexural strength and elastic modulus improved at low filler concentrations, reaching optimal performance at 5 wt.% PEEK, followed by a decline at higher loadings due to interfacial defects and particle agglomeration. Surface microhardness increased with PEEK content, whereas water sorption remained within ISO limits across all groups. In contrast, water solubility exceeded recommended thresholds at ≥5 wt.% PEEK, indicating compromised matrix integrity at higher filler loadings. Color stability was not significantly affected within the evaluated period. Overall, low-level PEEK incorporation (≤3–5 wt.%) provides a balanced improvement in mechanical performance and physicochemical stability. These findings highlight the importance of optimizing filler concentration in particulate-reinforced PMMA systems. From a materials engineering perspective, future work should focus on improving interfacial interactions through surface modification of PEEK particles (e.g., coupling agents such as ethylene glycol dimethacrylate, EGDMA), as well as incorporating microstructural characterization and long-term aging evaluation. These approaches will support the development of next-generation PMMA-based composites with enhanced performance and durability.

## Data Availability

The original contributions presented in this study are included in the article. Further inquiries can be directed to the corresponding authors.
